# Cavin-1 regulates caveolae-mediated LDL transcytosis: crosstalk in an AMPK/eNOS/ NF-κB/Sp1 loop

**DOI:** 10.18632/oncotarget.21944

**Published:** 2017-10-19

**Authors:** Xiang-Li Bai, Xiao-Yan Yang, Ju-Yi Li, Ye-Li, Xiong Jia, Zhi-Fan Xiong, Yu-Mei Wang, Si Jin

**Affiliations:** ^1^ Institute of Geriatric Medicine, Liyuan Hospital, Tongji Medical College, Huazhong University of Science and Technology, Wuhan, Hubei 430077, China; ^2^ Department of Clinical Laboratory, Liyuan Hospital, Tongji Medical College, Huazhong University of Science and Technology, Wuhan, Hubei 430077, China; ^3^ Department of Pharmacology, Hubei Key Laboratory of Drug Target Research and Pharmacodynamic Evaluation, Tongji Medical College, Huazhong University of Science and Technology, Wuhan, Hubei 430030, China; ^4^ Department of Endocrinology, Liyuan Hospital, Tongji Medical College, Huazhong University of Science and Technology, Wuhan, Hubei 430077, China; ^5^ Department of Nephrology, Union Hospital, Tongji Medical College, Huazhong University of Science and Technology, Wuhan, Hubei 430022, China

**Keywords:** cavin-1, low density lipoprotein transcytosis, nitric oxide

## Abstract

Caveolae are specialized lipid rafts structure in the cell membrane and critical for regulating endothelial functions, *e.g.* transcytosis of macromolecules like low density lipoprotein (LDL) *etc*. Specifically, the organization and functions of caveolae are mediated by structure protein (caveolin-1) and adapter protein (cavin-1). The pathogenic role of caveolin-1 is well studied; nevertheless, mechanisms whereby cavin-1 regulates signaling transduction remain poorly understood. The aim of this study was designed to explore the role of cavin-1 in caveolae-mediated LDL transcytosis across endothelial cells. We reported here that cavin-1 knockdown mediated by small interfering RNA (siRNA) caused a significant decrease of LDL transcytosis. Moreover, cavin-1 knockdown increased the activity of endothelial nitric oxide synthase (eNOS) and the production of nitric oxide (NO). Consequently, an eNOS inhibitor, N-Nitro-L-Arginine Methyl Ester (L-NAME), not only suppressed the activity of specificity protein (Sp1) and nuclear factor kappa B (NF-κB), but also inhibited both activities via activating adenosine 5‘-monophosphate- activated protein kinase (AMPK). In conclusion, we proposed an AMPK/eNOS/NF-κB/Sp1 circuit loop was formed to regulate caveolae residing proteins’ expression, *e.g.* LDL receptor (LDLR), caveolin-1, eNOS, thereby to regulate caveolae-mediated LDL transcytosis in endothelial cells.

## INTRODUCTION

Caveolae are defined as 50-100 nm flask-shaped invaginations of the plasma membrane [1, 2]. They are especially abundant in specific cell types such as endothelial cells, adipocytes, and muscle cells [3, 4]. Caveolae involve in a number of functions including endocytosis, transcytosis of macromolecules like LDL *etc* [4–6]. Caveolin-1 and cavin-1 are two essential structural components of caveolae [6, 7].

Caveolin-1 is a 21-22 kD integral membrane protein. It contains cytoplasmic N- and C-terminal domain that intercalate the membrane-association domain including the caveolin scaffold domain and the intramembrane domain [6]. The function of caveolin-1 has been intensively studied. Caveolin-1 mediates endocytosis and transcytosis of LDL in endothelial cells. A caveolin-1 deficiency prevents the transcytosis of LDL across endothelial cells [8]. We also previously reported that TNF-α up-regulates expression of caveolin-1 and LDLR via activating NF-κB, and consequently promotes LDL transcytosis across endothelial cells [9]. In addition, caveolin-1 maintains endothelial nitric oxide synthase (eNOS) in an inactive state [10], consequently limiting NO production [11, 12]. NO is an important gas signaling molecule. It not only regulates vascular tone and permeability, but also regulates activity of protein kinases and transcriptional factors. Caveolin-1 deficiency gives rise to a significant NO production, although the expression of eNOS is down-regulated in caveolin-1 siRNA-treated cells or in caveolin-1 knock out mice [13, 14].

Cavin-1 was originally thought as a polymerase I and transcript release factor (PTRF) to promote transcription. More recently, cavin-1 was found localized in caveolae and proved to be an important structure protein of caveolae. Cavin-1 is recruited to caveolae to stabilize caveolin-1 immediately when caveolin-1 oligomers settle down in the cell membrane [15]. The expression of cavin-1 and caveolin-1 are tightly linked; over-expression of cavin-1 lead to a increased expression of caveolin-1, whereas loss of cavin-1 result in a loss of caveolin-1 because of mis-location of caveolin-1 or enhanced lysosomal degradation of caveolin-1 [16]. Furthermore, genetic deletion of cavin-1 in mice was observed impaired caveolae formation [17, 18]. However, it is yet to be fully clarified whether or not cavin-1 plays any role in regulating LDL transcytosis and eNOS activity.

In present study, we tested the effect of cavin-1 on LDL transcytosis in human umbilical vein endothelial cells (HUVECs) transfected with cavin-1 siRNA. To further clarify the molecular mechanisms, the roles of the downstream protein kinases and transcriptional factors of NO, including AMPK, NF-κB and Sp1 were also invested. We found cavin-1 down-regulation inhibited LDL uptake and transcytosis, which were abrogated by eNOS/AMPK inhibitors. In addition, a significant increased NO production was observed; meanwhile, a decreased protein and mRNA expression of eNOS, caveolin-1 and LDLR was observed, which was associated with the increased NO level. NO-induced AMPK activation and NF-κB /Sp1 inhibition may coordinately contribute to these phenomena.

## RESULTS

### eNOS/AMPK signaling is involved in cavin-1 siRNA-suppressed LDL uptake and LDL transcytosis

**Figure 1:**
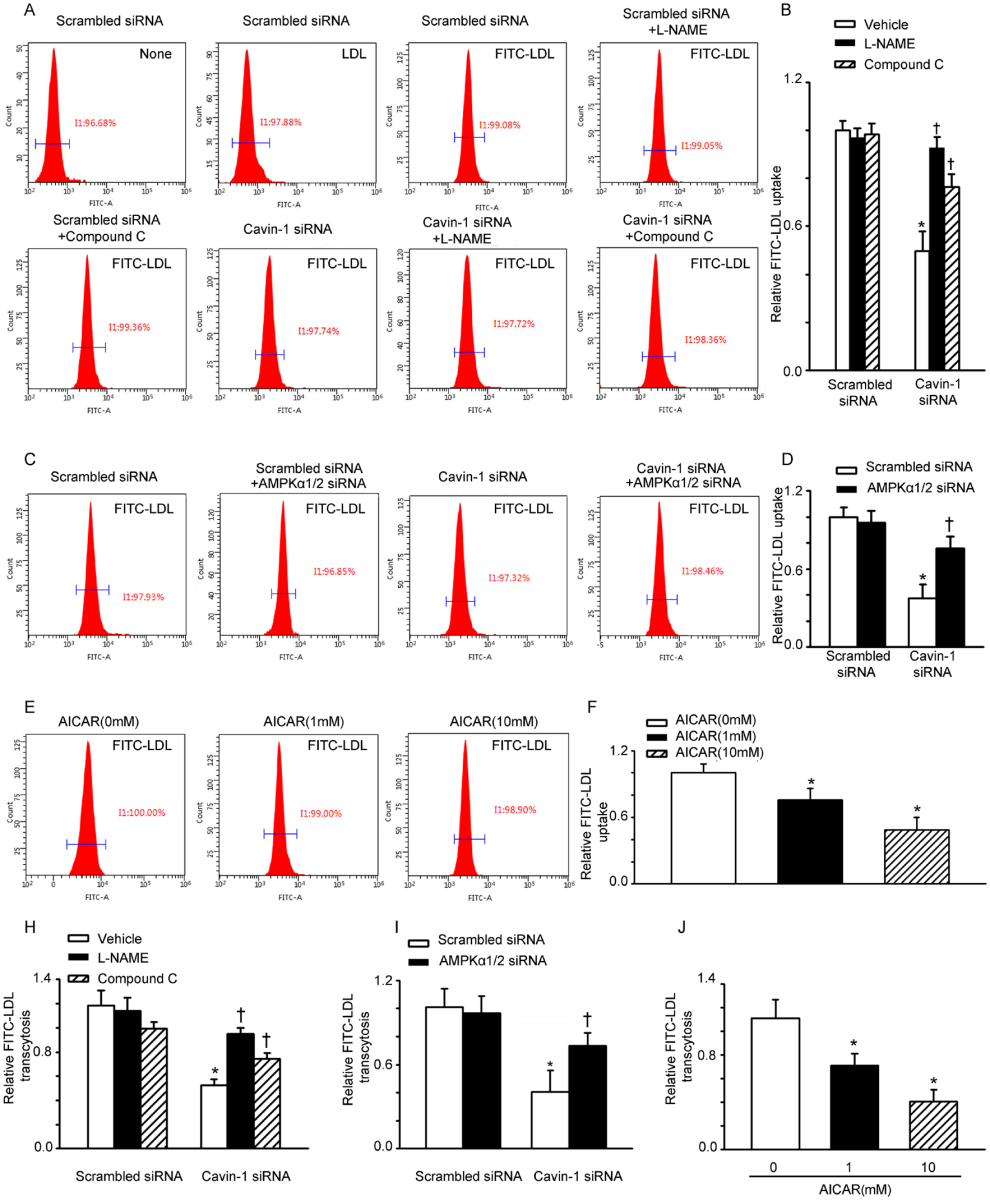
Cavin-1 siRNA suppressed LDL uptake and LDL transcytosis in HUVECs **(A, B)** Cells were transfected with scrambled siRNA or cavin-1 siRNA (20 nM) for 48 h, then incubated with L-NAME (50 μM) or Compound C (1 μM ) for 48 h. **(C, D)** Cells were transfected with scrambled siRNA (20 nM) or cavin-1 siRNA (20 nM) for 3 h, then co-transfected with scrambled siRNA (10 nM) or AMPKα1/2 siRNA (10 nM) for 45 h. **(E-F)** Cells were treated with indicated concentration of AICAR. (A, C, E) Flow cytometry images of FITC-LDL uptake in HUVECs incubated with FITC-LDL. (B, D, F) Summary bar graph showing the mean FITC-LDL fluorescent intensity in each group. **(H, I, J)** Quantitative summary of FITC-LDL transcytosis in HUVECs. * *P* <0.05 *vs*. scrambled siRNA or AICAR (0 mM); † *P* <0.05 *vs*. cavin-1 siRNA, *n*=4.

Several lines of evidence suggest that caveolae contribute to LDL transcytosis across endothelial cells [5, 7, 15]. However, is not clear whether cavin-1 can affect LDL transcytosis. The uptake of LDL by endothelial cells is an intermediate step of LDL transcytosis. We performed flow cytometry analysis to evaluate FITC-labeled LDL uptake in HUVECs. As shown in Figure 1A, after incubation with FITC-LDL, the mean fluorescent intensity of HUVEC, which reflects the level of LDL uptake, was significantly increased. Treatment with cavin-1 siRNA significantly reduced the mean fluorescent intensity, indicating a decreased LDL uptake. However, both the endothelial nitric oxide synthase (eNOS) inhibitor (L-NAME) and AMPK inhibitor (Compound C) increased the uptake of LDL, compared with that of cavin-1 siRNA-treated cells (Figure 1A and 1B). To evaluate the impact of AMPK on LDL uptake, AMPKα1/2 siRNA was used to specifically suppress the activity of AMPK. Knockdown of cavin-1consistently inhibited the uptake of LDL per cell, which was partially restored by AMPKα1/2 siRNA (Figure 1C and 1D). To further evaluate the impact of AMPK on LDL uptake, cells were incubated with 5-aminoimidazole-4-carboxamide-1-β-d-ribofuranoside (AICAR), a widely used an AMPK activator [19–22]. As shown in Figure 1E and 1F, treatment with AICAR decreased the uptake of LDL in HUVECs in a concentration-dependent manner. Next, we determined the level of LDL transcytosis across HUVECs by using an *in vitro* model of LDL transcytosis. As shown in Figure 1H and 1I, cavin-1 knockdown caused a significant inhibition of LDL transcytosis, which was completely abrogated by L-NAME and partially abrogated by AMPK inhibition (Compound C or AMPKα1/2 siRNA). In addition, treatment with AICAR decreased LDL transcytosis in a concentration-dependent manner (Figure 1J).

### Time course for caveolin-1 and eNOS expression after cavin-1 siRNA transfection

**Figure 2:**
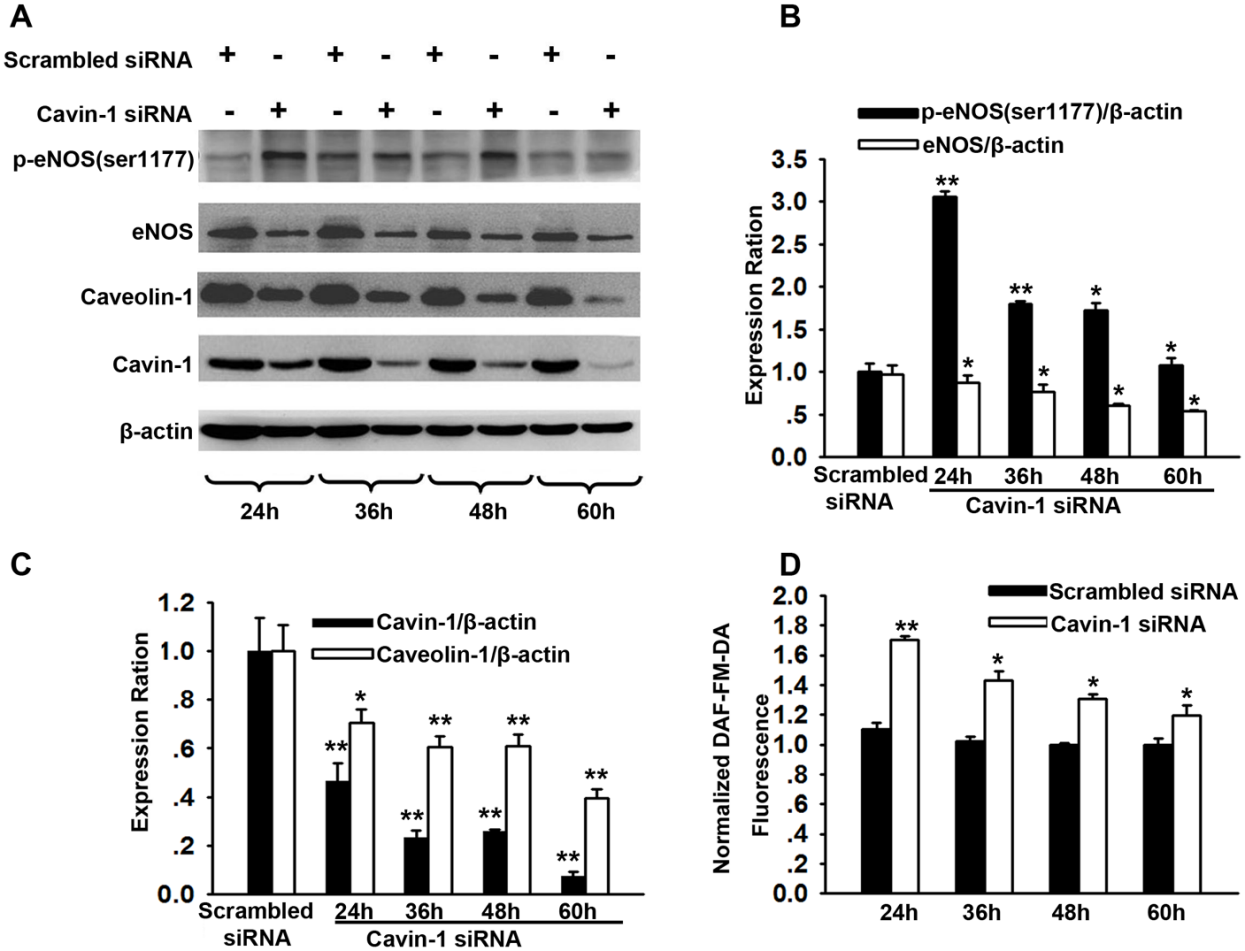
Time course for caveolin-1 and eNOS expression after cavin-1 siRNA transfection Cells were transfected with cavin-1 siRNA or scrambled siRNA (20 nM) for 24-60 h. **(A)** The representative western blots image shows the expressions of eNOS, caveolin-1 and cavin-1 at indicated time after siRNA transfection. **(B, C)** The protein levels of eNOS (B), caveolin-1 (C) and cavin-1 (C) were evaluated by immunoblotting. * *P* <0.05 or ^**^P <0.01 *vs*. Scrambled siRNA, *n*=4. **(D)** Cavin-1 siRNA enhanced NO production in HUVECs. HUVECs were transfected with cavin-1 siRNA or scrambled siRNA for indicated time and then loaded with DAF-FM-DA dye. After washing with PBS, fluorescence was measured. * *P* <0.05 or ^**^P <0.01 *vs*. Scrambled siRNA, *n*=4.

LDL transcytosis across endothelial cells is dependent on the function of caveolin-1 [1]. In the present study, we determined that treatment with cavin-1 siRNA inhibited the expression of caveolin-1 after cavin-1 siRNA transefection for 24 h, 36 h, 48 h, and 60 h, which was consistent with the findings presented in previous studies (Figure 2A and 2C) [16]. eNOS is the main regulator of endothelial function by producing NO. Caveolin-1 in caveolae directly interacts with eNOS and regulates nitric oxide (NO) production [12, 23]. NO not only affects vascular tone but also has an impact on gene expression [24]. We further explored the time course for eNOS activity and NO production after cavin-1 siRNA transfection at indicated time points. Compared with scrambled siRNA-treated cells, increased p-eNOS (Ser1177) expression and increased NO production were observed at 24 h, 36 h, 48 h, and 60 h after start of the cavin-1 siRNA transfection (Figure 2B and 2D). The data indicated that cavin-1 siRNA treatment may promote eNOS activation, which was similar to the effects caused by caveolin-1 deficiency [12]. Moreover, we also observed that treatment of cells with cavin-1 siRNA persistently decreased the expression of eNOS from 36 h after start of the transfection.

### Cavin-1 knockdown decreased the expression of caveolin-1, eNOS and LDLR

**Figure 3:**
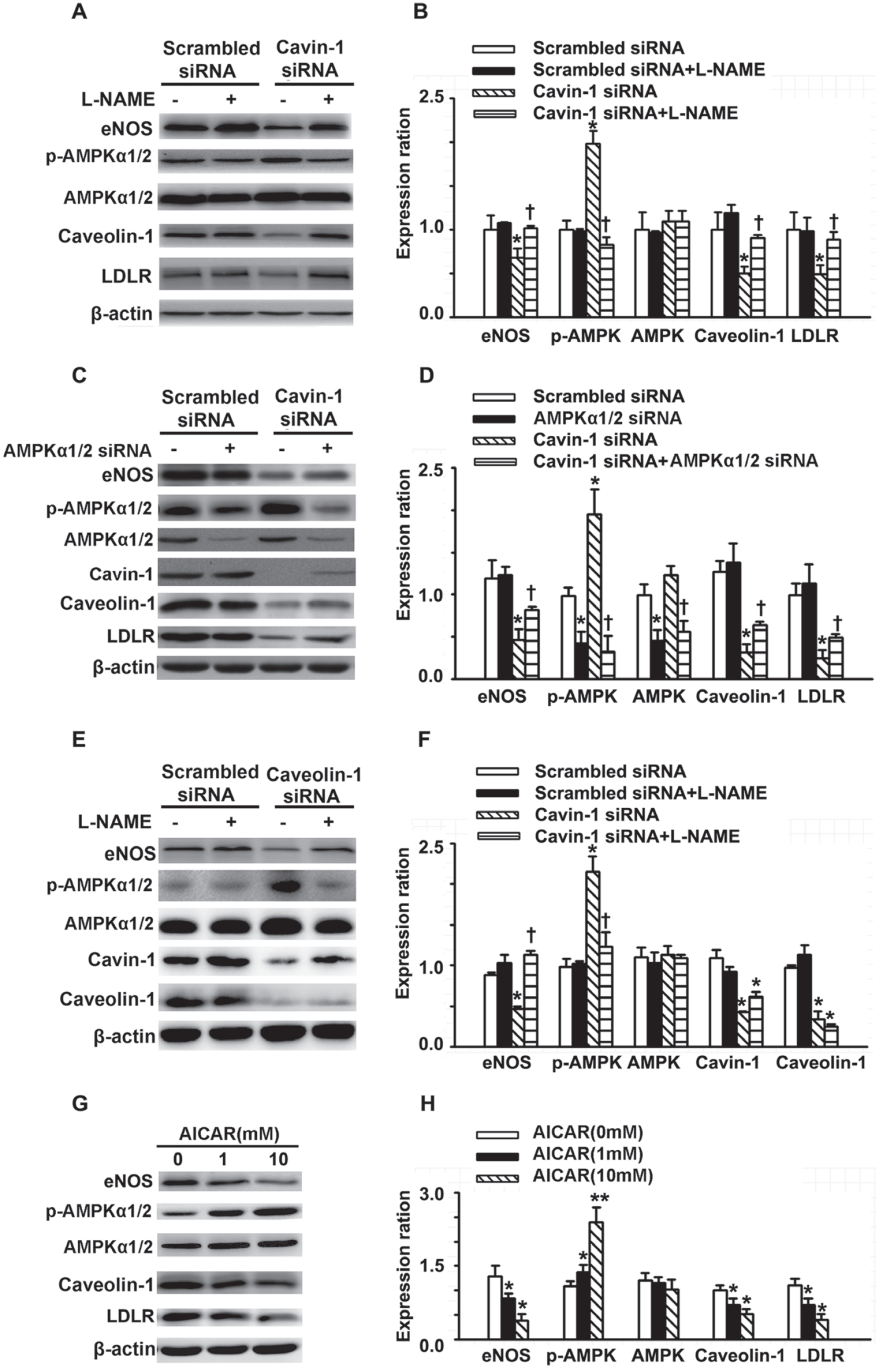
Cavin-1 knockdown decreased the expression of caveolin-1, eNOS and LDLR **(A, B)** Cells were transfected with scrambled siRNA or cavin-1 siRNA (20 nM) for 48 h, then incubated with L-NAME (50 μM) for 48 h. **(C, D)** Cells were transfected with scrambled siRNA (20 nM) or cavin-1 siRNA (20 nM) for 3 h, then cotransfected with scrambled siRNA(10 nM) or AMPKα1/2 siRNA (10 nM) for 45 h. **(E, F)** Cells were transfected with scrambled siRNA or caveolin-1 siRNA (20 nM) for 48 h, then incubated with L-NAME (50 μM) for 48 h. **(G-H)** Cells were treated with indicated concentration of AICAR. The protein level of cavolin-1, LDLR, AMPK and eNOS was evaluated by western blotting. (A, C, E, G) The representative images of western blotting. (B, C, D, F) The quantitative analysis of the expression of proteins. ^*^P <0.01 *vs*. scrambled siRNA or AICAR (0 mM), ^**^P <0.01 *vs.* AICAR (0 mM), *n*=4. † *P* <0.05 *vs*. Cavin-1 siRNA.

As mentioned above, knockdown of cavin-1 significantly induced NO production, but reduced eNOS expression. NO regulates gene expression in various cell types and species and induces S-nitration of transcription factors, such as NF-κB and subsequent inhibition of its activity [13]. In addition, NO is an endogenous activator of AMPK and functions via a guanylyl cyclase-mediated and Ca^2+^-dependent CaMKK pathway [25]. Moreover, it has been reported that AMPK and NF-κB /Sp1 directly or indirectly contribute in regulating the expression and activity of caveolae-residing proteins, such as eNOS, caveolin-1, and the LDL receptor (LDLR) [26–28]. We speculated that activating NO/AMPK signals are critical for cavin-1 to modulate the expression of caveolae-residing proteins, such as eNOS, caveolin-1, and LDLR. To elucidate the role of NO/AMPK signals on cavin-1 siRNA-reduced expression of caveolin-1, LDLR, and eNOS, we evaluated the protein expression of these caveolae-residing proteins in the presence of NO/AMPK signals inhibitors. First, L-NAME was used to inhibit the activities of eNOS. As shown in Figure 3A and 3B, knockdown of cavin-1 suppressed the expression of caveolin-1, LDLR, and eNOS, which was completely reversed by treatment with L-NAME. Furthermore, transfection with cavin-1 siRNA reduced the protein expression of caveolin-1, eNOS, and LDLR, which were partially abrogated by AMPKα1/2 siRNA (Figure 3C and 3D). Under physiological conditions, AICAR treatment reduced the expression of caveolin-1, eNOS, and LDLR (Figure 3G and 3H). Moreover, we also observed that the eNOS inhibitor L-NAME suppressed cavin-1 siRNA– or caveolin-1 siRNA–induced activation of AMPK, as indicated by the expression of p-AMPK (Thr172, Figure 3A-B and 3E-F).

### Cavin-1 siRNA inhibited the activity of NF-κB/Sp1, consequently reducing the mRNA expression of caveolin-1, eNOS and LDLR

**Figure 4:**
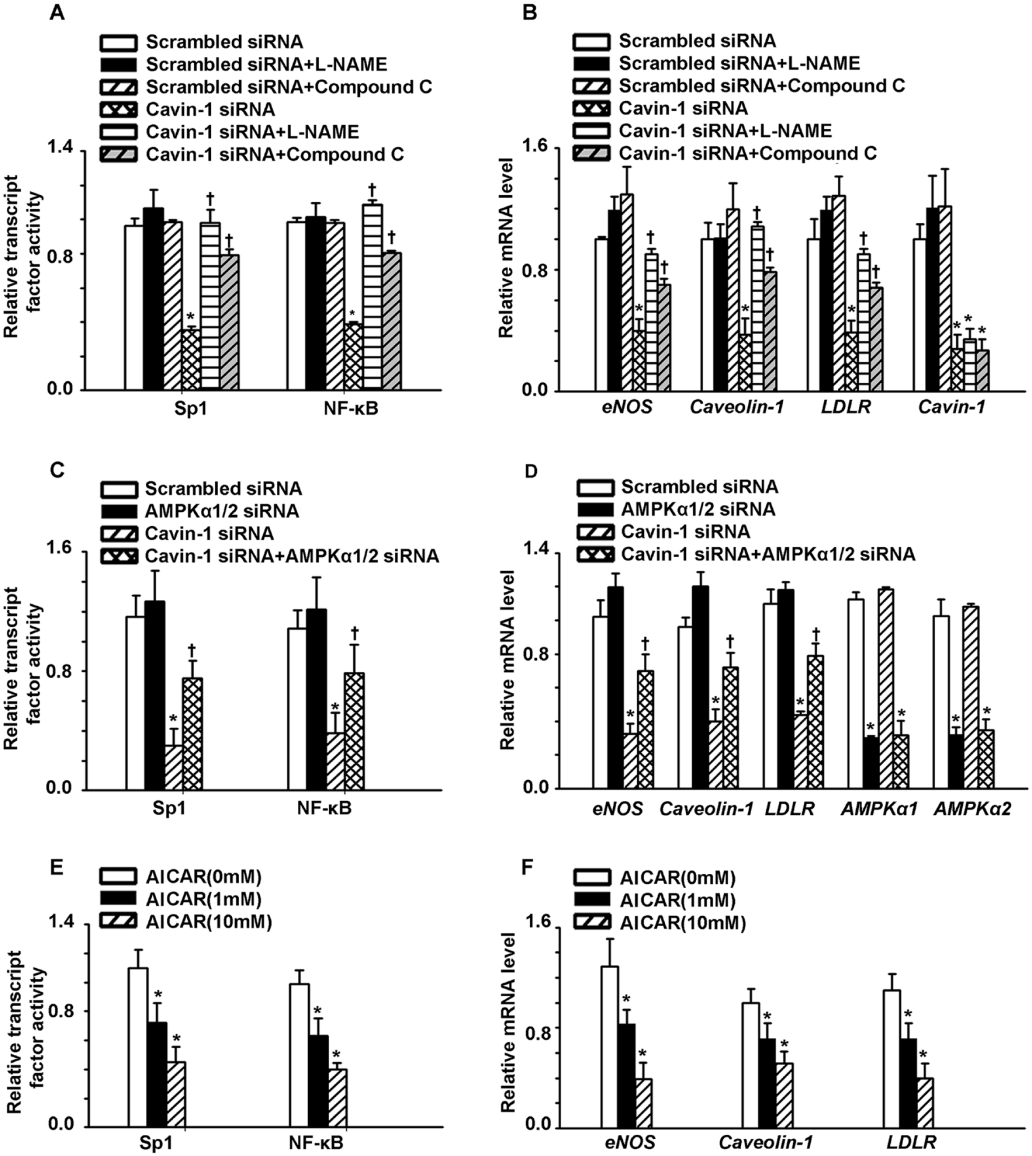
Cavin-1 knockdown inhibited the activity of NF-κB/Sp1, consequently reducing the mRNA expression of caveolin-1, eNOS and LDLR **(A, B)** Cells were transfected with scrambled siRNA or cavin-1 siRNA (20 nM) for 48 h, then incubated with L-NAME (50 μM) or Compound C (1 μM ) for 48 h. **(C, D)** Cells were transfected with scrambled siRNA (20 nM) or cavin-1 siRNA (20 nM) for 3 h, then cotransfected with scrambled siRNA (10 nM) or AMPKα1/2 siRNA (10 nM) for 45 h. **(E, F)** Cells were treated with indicated concentration of AICAR. (B, D, F) The mRNA level was evaluated by qPCR. (A, C, E) The activity of NF-κB or Sp1 was detected. * *P* < 0.05 *vs*. scrambled siRNA; † *P* < 0.05 *vs*. cavin-1 siRNA; *n*=4.

Previous studies have shown that NF-κB and Sp1 are involved in the transcription of LDLR, caveolin-1, and eNOS [29–32]. In addition, NO has been shown to suppress these transcription factors [31, 33–40]. Therefore, the increased levels of NO induced by cavin-1 knockdown may directly down-regulate NF-κB/Sp1 activity or indirectly down-regulate their activity via activating AMPK, thereby suppressing the transcription of LDLR, caveolin-1, and eNOS. Thus, we sought to evaluate the impact of cavin-1 knockdown-induced eNOS/AMPK signaling pathway activation on the activity of NF-κB/Sp1 and the expression of downstream genes, including LDLR, caveolin-1, and eNOS. As shown in Figure 4A, L-NAME up-regulated the activity of NF-κB/Sp1, when compared with cavin-1 siRNA-treated cells. Similarly, the reduced mRNA levels of LDLR, caveolin-1, and eNOS as caused by cavin-1 siRNA treatment were restored by treatment with L-NAME (Figure 4B). Since AMPK has been reported to inhibit the activity of NF-κB/Sp1 , Compound C and AMPKα1/2 siRNA were used to identify the role of AMPK activation in this process. As shown in Figure 4A and 4C, AMPK inhibition (as mediated by Compound C and AMPKα1/2 siRNA treatment), partially restored the decreased activity of NF-κB/Sp1 caused by cavin-1 siRNA. Moreover, cavin-1 siRNA reduced the mRNA levels of LDLR, caveolin-1, and eNOS, which were partially restored by AMPK inhibitors (Compound C and AMPKα1/2 siRNA) (Figure 4D). In addition, treatment with AICAR decreased the activity of NF-κB/Sp1, consequently inhibiting mRNA expression levels of caveolin-1, eNOS, and LDLR (Figure 4E and 4F).

## DISCUSSION

Expression of cavin-1 and caveolin-1 are closely linked [14, 16, 18, 23, 41]. The loss of fine balance is expected to result in impaired caveolae and dysfunction of caveolae-related signaling, *e.g.* LDL transcytosis and eNOS. In present study, we first demonstrated that cavin-1 is essential for caveolae-mediated LDL transcytosis in endothelial cells, and cavin-1 knockdown increased eNOS activity and NO production. Reacting to the increased NO, the activity of NF-κB/Sp1 is inhibited by NO or AMPK, consequently suppressed expression of caveolae-residing proteins such as LDLR, caveolin-1 and eNOS.

Cavin-1 is one of the essential components of caveolae, and is found most abundantly in endothelial cells [42–44]. Functionally, cavin-1 stabilizes caveolae by anchoring caveolin-1 to cytoskeleton via a C-terminal region. Changes of cavin-1 expression result in a comparable change of caveolin-1 expression [18, 44]. Indeed, cavin-1 deficiency leads to loss of morphologically identifiable caveolae [23]. Loss of cavin-1 release caveolae components including caveolin-1 into plasma and increase their lysosomal degradation [16]. Here, consistent with previous studies [45], we also found down-regulation of caveolin-1 expression in cavin-1 knockdown. The interaction of caveolin-1 with eNOS is intensively proved to maintain eNOS in an inactive state [46, 47]. We showed that knockdown of cavin-1 disrupted the association of caveolin-1 with eNOS, thereby enhanced eNOS activity and NO production (Figure 2B and 2D). However, we accidently found the expression of eNOS was significantly decreased from 36 h to 60 h (Figure 2B). Given that NO is an important gas signal molecule, it is necessary to ensure that it is produced the right amount in the right position. Shen *et al* reported that NO suppressed VEGF-induced increase in eNOS expression, indicating a physiological role for NO in feedback inhibiting eNOS expression [48]. Moreover, Isabella M. Grumbach confirmed that a negative feedback mechanism involving nitric oxide and NF-κB modulates eNOS transcription.Thus, there may be a negative feedback mechanism that down-regulates eNOS expression to limit the NO production when responding to an increased NO level caused by cavin-1 knockdown.

It is reported that NO can regulate the transcription via direct s-nitration of transcript factors [13]. We assume that NO may mediate the decrease in eNOS expression induced by cavin-1 knockout. Hence, we tested the mRNA and protein expression of eNOS, caveolin-1 and LDLR with or without L-NAME (an eNOS inhibitor) in HUVECs transfected with cavin-1 siRNA. As we expected, L-NAME treatment up-regulated the decreased mRNA and protein expression of eNOS, caveolin-1 and LDLR induced by cavin-1 or caveolin-1 knockdown. Previous study has reported that AMPK is also a down-stream signaling molecule of NO [37]. Thus we also measured the activity of AMPK and found that L-NAME treatment inhibited AMPK activation induced by cavin-1 or caveolin-1 knockdown. Furthermore, cavin-1 siRNA suppressed expression of eNOS, caveolin-1 and LDLR, which were partially restored by AMPK inhibitors (Compound C and AMPKα1/2 siRNA). In addition, AICAR, a tool drug employed to activate AMPK, suppressed the expression of eNOS, caveolin-1 and LDLR in physiological condition. In summary, the loss of cavin-1 inhibited the expression of eNOS, caveolin-1 and LDLR, while activating AMPK by increasing eNOS-derived NO.

**Figure 5:**
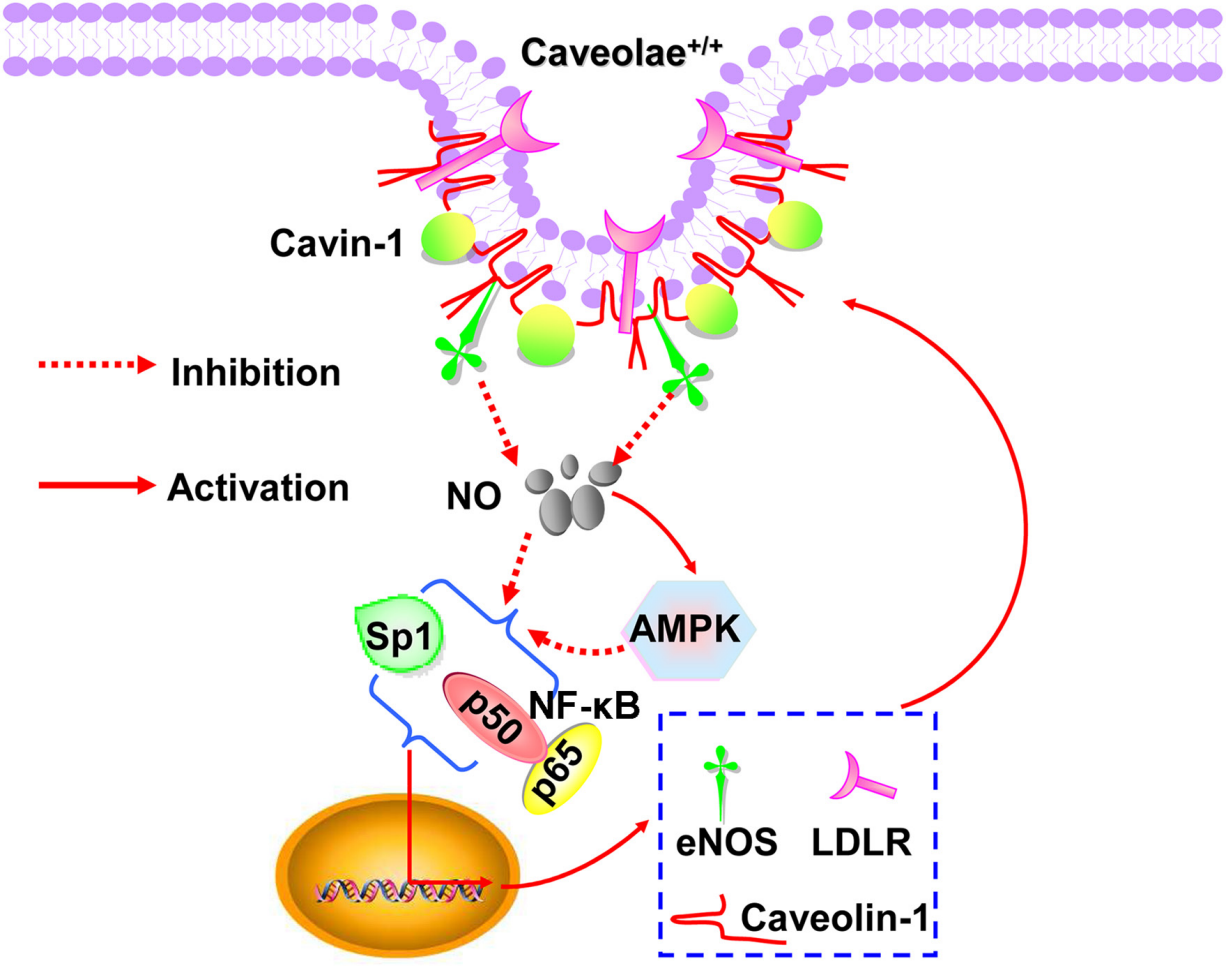
Schematic diagram summarized the molecular mechanism underlying the cavin-1-mediated regulation of downstream proteins

NF-κB /Sp1, two transcript factors, mediate the transcription of eNOS, caveolin-1 and LDLR [9, 13, 40, 49]. NO cannot only suppress the activity of NF-κB /Sp1, but also inhibit both activities via activating AMPK [28, 50]. Here, we showed that cavin-1 siRNA suppressed the activity of NF-κB/Sp1, which was abrogated by L-NAME. However, AMPK inhibition (as mediated by Compound C and AMPKα1/2 siRNA treatment), partially restored the decreased activity of NF-κB/Sp1 caused by cavin-1 siRNA. As summarized in Figure 5, all of these dated elucidated that cavin-1 knockout increased NO production, which then induced an AMPK/eNOS/NF-κB/Sp1 circuit loop to regulate caveolae residing proteins’ expression, *e.g.* LDLR, caveolin-1, eNOS, thereby to modulate caveolae-mediated LDL transcytosis in endothelial cells.

## MATERIALS AND METHODS

### Primary culture of human umbilical vein endothelial cells

The collection of human umbilical cords was approved by the Ethics Committee of Tongji Medical College, Huazhong University of Science and Technology (Wuhan, China) and conducted in accordance with the Declaration of Helsinki (2008) and all applicable national and local regulations. All subjects provided written informed consent prior to the initiation of the study. HUVECs were isolated as described [51]. The cells were cultured in endothelial cell medium (ECM, Sciencell, Carlsbad, CA) at 37°C in an incubator with a humidified atmosphere of 5% CO_2_. Cells were used between passages 2 and 4.

### RNA interference

HUVECs grown to confluence in 40%∼50% were transfected with 20 nM cavin-1 siRNA (GGAGGTTGAGGAGGTTATT), caveolin-1 siRNA(AACGAGAAGCAAGTGTACGAC) or AMPK α1/2 siRNA (Santa Cruz Biotech-nology, CA, USA) using Hyperfect transfection reagent (Qiagen, Hilden, Germany). siRNAs were synthesized by RIBOBIO (Guangzhou, China). To evaluate the effect of NO, L-NAME at the concentration of 50 μM was added at the beginning of siRNA transfection, Complete medium containing 50 μM L-NAME was replenished every 24 h. AICAR (Selleckchem, Houston, TX, USA).

### Western blot assay

Western blot assay was carried out as described previously [52]. The following primary antibodies were used at 1:1000 dilutions: rabbit anti-p-eNOS ser1177, anti-eNOS, anti–p-AMPKα thr172, anti-AMPKα, anti-caveolin-1, and anti-β-actin purchased from Cell Signaling Technology (Beverly, MA); Anti-cavin-1 and anti-LDLR purchased from Proteintech (China). The goat anti-rabbit and goat anti-mouse secondary antibodies from Abbkine (Redlands, CA, USA) were used at 1:10 000 dilution.

### NF-κB and Sp1 activity assay

NF-κB and Sp1 activity was assayed by an ELISA-based method as described previously [53]. Total cell extracts were collected and quantified with BCA reagent (Pierce, Rockford, IL). The cell extracts were incubated in a streptavidin-coated 96-well plate (Roche Diagnostics Corporation, Indianapolis, IN 46256), which had been coated with 3’end biotinylated oligonucleotide containing the conservative binding site NF-κB or Sp1. The sequences were shown in Supplementary Table 1. Activated transcription factors from extracts specifically bound to the respective immobilized oligonucleotide. NF-κB or Sp1 activity was detected with NF-κB or Sp1 antibody (1:500, Proteintech, China) and secondary antibody conjugated to horseradish peroxidase (1:10 000, Abbkine, CA). NF-κB and Sp1 activity was finally determined as absorbance values measured with a microplate reader at a wavelength of 450 nm.

### RNA extraction and quantitative PCR

Total RNA was extracted from HUVECs using the TRIzol reagent (Invitrogen), and 2 μg of RNA was used to synthesize the first strand cDNA using RevertAid First Strand cDNA Synthesis Kit (Thermo Scientific). cDNA (2 μl) was amplified using specific primers (Supplementary Table 1) and SYBR Green qPCR kit (TOYOBO, Osaka, Japan).The specific primers are listed in Supplementary Table 1. Expression levels were normalized to β-actin.

### Measurement of intracellular NO production

The cell-permeable dye 4,5-diaminofluorescein diacetate (DAF-FM-DA, Beyotime Institute of Biotechnology, China) was used to measure the intracellular NO levels. The experiment was conducted as described previously [54].

### LDL uptake in HUVECs

FITC-LDL was prepared as described previously [55]. HUVECs were seeded on 12-well plate (Roche Diagnostics Corporation, Indianapolis, IN 46256) and were transfected with specific siRNA for 45h. And then, the transfected cells were incubated with serum-free Opti-MEM containing FITC-LDL (50 μg/ml) for 3 h. Cells were harvested using 0.125% typsin (with out EDTA) and the uptake of LDL were measured by flow cytometry (Mindry, Bricyte E6). Meanwhile, background fluorescence of HUVEC treated with naïve LDL was subtracted from the mean FITC-LDL fluorescent intensity of each sample, representing the uptake of FITC-LDL.

### Measurement of LDL transcytosis

The amount of LDL transcytosis was measured as described previously [55]. Briefly, HUVECs were seeded on polyester membrane of costar transwell (6.5 mm diameter and 0.4 μm pore size) and the integrity of the cell monolayer was tested by a method described previously [56]. Two inserts of cell monolayers with equal integrity were assigned into the same group: the non-competitive insert and the competitive insert. HUVECs grown to confluence in 30-40% were transfected with siRNA for 45h. The non-competitive insert was incubated with FITC-LDL (50 μg/ml) for 3h to determine the total amount of trans-endothelial LDL. Paracellular transport was determined by incubation with 50 μg/ml FITC-LDL and 6-fold excess unlabeled LDL (300 μg/ml) in competitive insert. Samples were then collected from the outer chambers and further dialyzed against PBS to remove the free FITC due to degradation or metabolism in the cell. The FITC fluorescent intensity was measured by a fluorescence spectrophotometer (Tecan, Ininite F200PRO) with excitation and emission wavelengths of 490nm and 520nm, Meanwhile, background fluorescence of serum-free Opti-MEM was subtracted from the value of each sample. The amount of LDL transcytosis is the difference in the fluorescent intensity between the non-competitive insert and the competitive insert.

### Statistical analysis

All data were expressed as the mean± SEM from at least three separate experiments. Unpaired Student *t*-tests with Bonferroni correction were used to analyze individual group statistical comparisons, and one-way ANOVA with *post hoc* testing was used to evaluate multiple group comparisons. Statistical significance is defined as *P* <0.05.

## SUPPLEMENTARY MATERIALS TABLE


