# Supplementary material for: Cavin-1 regulates caveolae-mediated LDL transcytosis: crosstalk in an AMPK/eNOS/ NF-κB/Sp1 loop
Source: Oncotarget. 2017 Oct 19;8(61):103985–95. doi: 10.18632/oncotarget.21944 (PMC5732781; doi:10.18632/oncotarget.21944)
Supplement: Supplementary file 1 [file oncotarget-08-103985-s001.pdf]

Cavin-1 regulates caveolae-mediated LDL transcytosis: crosstalk in an AMPK/eNOS/ NF-κB/Sp1 loop

SUPPLEMENTARY MATERIALS

Supplementary Table 1: Oligonucleotide/primer sequence used for NF-κB and Sp1 activity assay or PCR

Oligonucleotide/Primer	Sequence
Sp1 consensus-binding	5'-ATTCGATCG GGGCGG GGC GAG C-(C)34-C-3'
oligonucleotide	3'-TAA GCT AGC CCC GCC CCG CTC G-5'
NF-κb consensus-binding	5'-AGT TGA GGG GAC TTT CCC AGGC--C-(C)34-C-3'
oligonucleotide	3'-TCA ACT CCC CTG AAA GGG TCC G-5'
eNOS forward primer	5'- TGGTCAACTATTTCCTGTCCCCG-3
eNOS reverse primer	5'- TTGTCGCCTTCACTCGCTTCG -3'
LDLR forward primer	5'-TGTCGTGTGTGTTGGGATGGG-3
LDLR reverse primer	5'- TGGAAGCACTAGGTGGGCGG -3'
Caveolin-1 forward primer	5'- AGAACCAGAAGGGACACACAG -3'
Caveolin-1 reverse primer	5'- AGGAAAGAGAGAATGGCGAAG -3'
Cavin-1 forward primer	5'- CATTGGTTTCCTTGTCCTTGGG-3'
Cavin-1 reverse primer	5'-GAGGGGCTGTTCTTGCTTCTG-3'
AMPKα1 forward primer	5'-AGCCCACCTGATTCTTTTCTT-3'
AMPKα1 reverse primer	5'-GCCATTTTGCTTTCCTTACAC-3'
AMPKα2 forward primer	5'-TGGTGTTATCTTGTATGCTCT-3'
AMPKα2 reverse primer	5'-TCATGCTCTCTTATGTCTTTG-3'
β-actin forward primer	5'- CCTTCCTGGGCATGGAGTC-3'
β-actin reverse primer	5'- TGATCTTCATTGTGCTGGGTG-3'
